# Social determinants of low uptake of childhood vaccination in high-risk squatter settlements in Karachi, Pakistan – A step towards addressing vaccine inequity in urban slums

**DOI:** 10.1016/j.jvacx.2023.100427

**Published:** 2023-12-30

**Authors:** Shifa Salman Habib, Shehla Zaidi, Atif Riaz, Hasan Nawaz Tahir, Lala Aftab Mazhar, Zahid Memon

**Affiliations:** a Department of Community Health Sciences, Aga Khan University, Stadium Road, P. O. Box 3500, Karachi 74800, Pakistan; b Aga Khan University (International), AKU-UK, Aga Khan Centre, 10 Handyside Street, London N1C 4DN, UK

**Keywords:** Childhood vaccination, Penta-3, Urban-slums, Vaccine inequity

## Abstract

**Background:**

Routine vaccination has remained historically low in major urban pockets of Pakistan, and now lags behind rural vaccination rates. Grossly insufficient publicly funded primary healthcare infrastructure, heterogeneous mix of providers and multi-ethnicity of populations pose challenges in the delivery of essential health services. This paper ascertains factors associated with uptake of routine childhood vaccination, using Pentavalent-3 vaccine, as a proxy indicator for completion of age-appropriate vaccines in urban slums of Karachi, at high risk of Polio and vaccine preventable disease outbreaks.

**Methods:**

Data was drawn from baseline assessment of an urban immunization delivery pilot project in urban slums of Karachi, Pakistan. The study sample comprised of 2,097 households with children aged 4–12 months, sampled through a cross-sectional cluster survey, applying a structured questionnaire. Multivariable logistic regression was used to determine the association between Penta-3 vaccination, as the outcome variable, and predictor variables including socio-demographic characteristics and healthcare access factors.

**Results:**

The findings showed that the likelihood of being immunized with Penta-3 was higher for non-Pashtun ethnicity [adjusted odds ratio (aOR) 1.69; 95% CI 1.33–2.14], children of educated mothers, secondary or higher [aOR 2.95, 95% CI 2.34–3.71], and those whose fathers were formally employed (aOR 1.53; 95% CI 1.19–1.97). No association was seen by gender of child [aOR 0.89; 95% CI 0.73–1.08], and place of new born delivery [aOR 1.01; 95% CI 0.83–1.24].

**Conclusion:**

Pockets of critically low under-vaccinations within the urban slums of Karachi are associated with Pashtun ethnicity, distance to the vaccination centre, lack of mothers’ education and lack of stable family income as in the case of unemployed and daily wage-earning fathers. Recognition of these factors is required in designing contextually appropriate strategies to address vaccine inequity in urban settings.

## Highlights

• The rate of completed childhood immunization up to Penta-3, is much lower in low-income urban settlements, than previously reported district wide and province-wide figures.
• There are critical missed encounters for vaccination within urban health facilities, being utilized for new-born deliveries.
• Likelihood of missed vaccinations was seen amongst children of Pashtun ethnicity, and those whose mothers lack formal education, fathers are unemployed or are daily wage earners, and reside at a distance of more than 2 km from the vaccination centres.

## Introduction

Routine childhood immunization is a global mandatory intervention for reducing child morbidity and mortality, however 25 million infants missed age-appropriate childhood vaccination in 2021 [1]. Five countries – Pakistan, Nigeria, India, Democratic Republic of Congo and Ethiopia - account for 50 % of all children globally, who missed out on essential birth vaccinations in 2020 [2]. While there has been improvement in rural vaccination rates, chronic challenges in the delivery of routine immunization services are seen in urban areas [3]. Public sector primary care infrastructure to deliver vaccine services is sparse in low-income urban areas [4], compounded by increased risk of vaccine preventable disease due to over crowdedness, inadequate sanitation [5] as well as vaccine hesitancy challenges due to multi-ethnic populations and influx of migrants [6].

Pakistan, is the fifth populous country [7], having a large birth annual cohort of 5 million infants and one of the highest infant mortality rates of 56 per 1000 live births [8]. Pakistan’s progress in immunization is stalled by low vaccination uptake in urban slums, leading to vaccine coverage disparities seen between urban and rural areas. The megacity of Karachi, is the largest urban centre of Pakistan, with a population of over 20 million, of which 46 % (7 million) reside in urban squatter settlements [9]. Karachi’s low-income areas are at high risk of polio transmission and eight out of 39 super-high risk union councils of Pakistan are located in Karachi [10]. Drop-out rates for childhood vaccination are high in these union councils with coverage of polio falling from 83 % at zero dose to 68.3 % by the third dose, whereas Penta3 coverage stands at 68.2 % as per immunization pooled registry, making the most populous city vulnerable to Vaccine Preventable Disease (VPD) outbreaks within and across provincial borders.

The World Health Organization (WHO) promotes targeted interventions for children residing in marginalized urban communities to reduce vaccine inequities [3] whereas Global Alliance for Vaccines and Immunization (GAVI) emphasizes bridging inequalities as a critical focus in achieving vaccinations for all by 2030 [11]. Immunization planning in Pakistan, in alignment with the global strategies, is re-purposing towards a targeted focus on immunization coverage in urban slums so as to reach the last mile with vaccinations. Global literature underscores the importance of sociodemographic factors such as religion, ethnicity, age and education of the parents, employment status, socio-economic condition, access to healthcare facility, and gender of the child have been identified as important predictors of immunization uptake [12], [13], [14], [15]. Within Pakistan’s context, the generation of local quality evidence on the drivers of routine childhood immunization Pakistan’s urban squatter settlements is required to inform the timely design and roll-out of targeted interventions.

This paper is based on evidence from an ongoing implementation research pilot jointly hosted by the Expanded Program of Immunization (EPI), Sindh and the Aga Khan University for addressing vaccine inequities in Karachi’s urban squatter settlements designated as high risk for polio. The paper draws on primary immunization coverage data from high defaulter and refusal areas within designated high-risk union councils with the objective of ascertaining sociodemographic and health access factors that have a significant impact on incomplete vaccinations in children under one year of age. Findings are interpreted and compared with global evidence base, and lessons drawn for building contextualized and calibrated strategies to address specific inequities in immunization uptake in marginalized urban communities.

This study uses uptake of the third dose of pentavalent vaccine (penta-3) (diphtheria, pertussis, tetanus, hepatitis B, haemophilus influenza type B); as a proxy indicator for completion of age-appropriate vaccinations. Penta-3 for children is an important indicator for assessing performance and utilization of an immunization programme because it mirrors the completeness of a child’s immunization schedule [16]. Increased coverage of Penta-3 was a key target of our implementation research pilot.

## Methodology

### Study setting & design

Karachi, with a population of 20 million, is administratively spread over 7 districts and 178 union councils (UCs). Union councils are the lowest public administrative tier for service delivery in Pakistan. A total of 8 Super-High Risk Union Councils (SHRUCS), out of a total of 39, that are under surveillance for polio transmission, are located in the mega city of Karachi, whereas an additional twenty-seven union councils are declared High Risk Union Councils (HRUCs). Data was drawn from baseline assessment, conducted as part of establishing the larger implementation pilot project on engaging private providers to supplement routine immunization services at 8 high risk union councils of Karachi. These union councils reflect the typical socioeconomic profile of low-income areas of Karachi comprising of multi-ethnic, low-income populations with low literacy and poor living conditions. A cross-sectional community survey of households was conducted of eligible households having children aged 4–11 months during 2022.

### Population

The study population included parents or caregivers having at least one child aged 4–11 months weeks of age and having resided in the study polygon for at least six months. Either mothers or fathers were eligible for participation, or routine caregiver in the absence of parents.

### Sample size and sampling technique

Baseline survey data of the larger implementation research pilot was analyzed to identify significant co-variates associated with Penta-3 uptake. The larger study was powered to measure a 20 % increase in Penta-3 over a two-year period. The baseline survey had a sample of 2097 children, calculated on the basis of Penta-3 coverage of 68 % among children under 1 year in Karachi, taken from the official government survey statistics (MICS Sindh 2018–19 for Karachi), a margin of equivalence ranging between 10 % and 20 % and the actual difference ranging from 5 % to 10 %. The two-sided level of significance was assumed to be 5 %.

The study universe comprised of 18 population polygons within the 8 HRUCs, that had the highest number of defaulter children as per data from the government immunization registry. Each polygon comprised of 25,000 households and had defined list of households. We used systematic sampling to enroll eligible households in the survey, applying WHO cluster sampling approach whereby clusters of 7 households were sampled from the 18 polygons [17]. The number of clusters to be sampled from each polygon was determined using probability proportional to size method, and a total of 968 clusters were sampled. First household from each cluster was randomly selected using spin of a pen and checked for eligibility. From first eligible household, every third household was checked for eligibility and was enrolled if eligible. In case of non-eligible household, the next household was checked for eligibility and the process continued until the cluster was completed. For every new cluster, a different direction was taken from the centre of polygon using random spin of pen.

### Data collection

A pre-tested, close-ended questionnaire, adapted from WHO tool for immunization coverage, was used to collect data from parents/ caregivers on immunization status, background characteristics and health seeking behavior from May to July 2022. Data were collected by trained research assistants recruited from local areas to gain respondent access and trust. After explaining the research purpose and seeking consent for participation, parents/caregivers of eligible children were interviewed in person using digital versions of the questionnaire and data collected on android tablets using open-source platform of EpiCollect 5. To ensure data quality, 10 % of the surveyed households were randomly selected for re-interview and spot observations during data collection.

### Dependent variable

According to the EPI schedule most of the vaccines are completed by the age of 14 weeks, hence Penta-3 administered at 14 weeks (4 months) was taken as a proxy for vaccination compliance in children under 1 year of age. Penta-3 vaccination status was taken as the dichotomous outcome variable with two categories:

1. Penta-3 immunized: a child who received a dose of BCG, four doses of OPV (OPV0, OPV1, OPV2, and OPV3), and three doses of pentavalent vaccine.
2. Penta-3 partially immunized: a child who missed any of the previously mentioned vaccines.

Vaccination status was be assessed through vaccination cards and verbal history from caregiver. There were five responses for each of these variables, namely, “no,” “don't know,” “yes- card observed,” and “yes-reported by mother.” The responses “no” and “don't know” were recoded as 0 (vaccine not received), whereas yes- card observed,” and “yes-reported by mother were recoded as 1 (vaccine received). Box 1 provides list of vaccines provided through EPI, Pakistan

Box 1 **: Schedule of Childhood Vaccination under Expanded Programme of Immunization, Pakistan.**

**Table:** 

Age	Vaccines
Birth	OPV 0, BCG, Hep-B
6 weeks	OPV I, Penta I, PCV I, Rota I
10 weeks	OPV II, Penta II, PCV II, Rota II
14 weeks	OPV III, Penta III, PCV III, IPV-1
9 months	MR 1, Typhoid conjugate Vaccine, IPV 2
15 months	MR 2

OPV = oral polio vaccine; BCG = Bacille Calmette–Guérin; Penta = pentavalent vaccine (diphtheria, pertussis, tetanus, hepatitis B, Haemophilus influenza type B); PCV = pneumococcal conjugate vaccine valent 10; IPV = injectable polio vaccine, MR = measles, rubella.

### Independent variables

Independent or explanatory variables were selected based on review of relevant literature from Pakistan and the region. The independent variables included participant characteristics that is gender of the child, education of the mother and father’s occupation. Mother’s education which was measured at five levels as described by the education department, none (not attended any formal or non-formal education including mosque based Madrasa education), Primary (up till Grade 8), Secondary (up till Grade 10), Higher Secondary (Grades 11–12), and University level (Bachelor’s, and Postgraduate degrees). The father’s occupation was defined as unemployed/daily wagers, government job, private job and self-employed and others. The independent variables also included health access and utilization characteristics that is place of delivery defined as private hospital or maternity home, government facility and home-based delivery, and distance to the nearest EPI centre (<2 km and > 2 km).

### Statistical analysis

Data analysis was performed in Stata version13 (College Station, TX: Stata Corp LP). Frequencies were generated for sociodemographic and baseline characteristics of the households. Cramer’s V test was run to assess multicollinearity between the correlates to avoid its negative effect on the multivariate analysis. Univariate and multivariate logistic regression was run to identify predictors of Penta 3 vaccination*,* and the adjusted odds ratio with 95 % CI were calculated. A Cramer’s V co-efficient of 0.8 and above was taken to show multi collinearity between the variables. A p-value of less than 0.05 was considered to be statistically significant..

### Ethical considerations

The study received approval from Ethical Review Committee (ERC) of Aga Khan University Pakistan (ERC number 2022–7079-20320). Participation in the survey was voluntary and written consent for study participation was obtained from parents/caregivers of all study participants. Data was stored with encryption and password protection and fully anonymized using unique identifiers.

## Results

### Descriptive statistics on sociodemographic characteristics and immunization status

A total of 6,775 households were approached for the survey, of which 2,097 were eligible for participation and were enrolled in the survey. Among the enrolled participants, 30.8 % (n = 645) children had received the Penta-3 vaccine. Further descriptive analysis of the 2,097 children is provided in Table 1. Among the participants, 51.6 % (n = 1,082) were males. Incomplete Penta-3 vaccinations were mainly seen amongst Pashtun (36.3 %), followed by Seraiki (17.4 %) ethnic groups, several of whom comprise of in-country migrants. The highest rate of Penta-3 vaccination was found among the Urdu speaking group with 35.3 %.

**Table 1:** Sociodemographic characteristics of study participants, children aged 4 to 11 months, by Penta-3 vaccination status, in urban settlements of Karachi, Pakistan, May to July 2022.

	**Vaccination status for Penta-3 vaccine**					
	**Vaccinated**		**Unvaccinated**		**Total**	
	**N**	**%**	**n**	**%**		
**Gender of child**						
Male	342	53.0	740	51.0	1,082	51.6
Female	303	47.0	712	49.0	1,015	48.4

**Ethnicity**						
Urdu	221	34.3	232	16	453	21.6
Pashto	140	21.7	527	36.3	667	31.8
Sindhi	119	18.5	207	14.3	326	15.6
Punjabi	48	7.4	73	5.	121	5.8
Baluchi	20	3.1	66	4.5	86	4
Siraiki	68	10.5	253	17.4	321	15.3
Others	29	4.5	94	6.5	123	5.9

**Place of delivery**						
Private hospital /maternity home	293	45.4	526	36.2	819	39.1
Government hospital	91	14.1	275	19.0	366	17.5
Community-based	261	40.5	651	44.8	912	43.5
						

**Distance to EPI centre**						
<2km	378	58.6	659	45.4	1,037	49.5
>2km	260	40.3	628	43.3	888	42.3
Don’t know	7	1.09	165	11.4	172	8.2

**Mother’s education**						
No education/ Home/Madarsa education	303	46.8	1,089	75.0	1392	66.3
Primary	60	9.3	98	6.8	158	7.5
secondary & Higher secondary	249	38.6	240	16.5	489	23.3
University and beyond	33	5.1	25	1.7	58	2.8

**Father’s occupation**						
Unemployed or Daily wagers	271	42.0	774	53.3	1045	49.8
Government Job	16	2.5	9	0.6	25	1.2
Private job	185	28.7	257	17.7	442	21.1
Self-employed	173	26.8	412	28.4	585	27.9

Among mothers of children who missed Penta-3 vaccination, 75 % had received no formal school education, whereas this proportion for vaccinated children was nearly 30 percentage points lower (46.8 %). In terms of father’s occupation, 53 % of the defaulters’ fathers were unemployed or engaged as daily wagers (Table 1).

A majority of the children in our study were born at private hospitals, among both vaccinated (34.6 %) and unvaccinated (26.7 %) groups. It was also found that nearly half of the study population (1,037 children) was residing within 2 km of a vaccination centre. However, a large proportion (63.5 %) among these did not receive the Penta-3 vaccination (Table 1).

### Factors associated with Penta-3 uptake

Table 2 shows the Cramer’s V coefficients to assess collinearity among covariates of Penta-3 uptake included in the analysis. According to these coefficients, no collinearity was found between the covariates (all co-efficients < 0.8), hence there was no impact on the multiple regression model. The results of the logistic regression analysis are presented in Table 3. The findings of the final adjusted model reveal that Penta-3 uptake was significantly associated with ethnicity, mother’s education status, father’s occupation and distance to the EPI centre.

**Table 2:** Cramer’s V co-efficients to assess collinearity between the socio-demographic co-variates for Penta-3 vaccination in children aged 4 to 11 months in urban settlements of Karachi, Pakistan.

	**Gender**	**Ethnicity**	**Place of delivery**	**Distance to the EPI centre**	**Mother's education**	**Father's occupation**
**Gender**	X					
**Ethnicity**	0.0664	X				
**Place of delivery**	0.0742	0.1323	X			
**Distance to the EPI centre**	0.0472	0.1798	0.1949	X		
**Mother's education**	0.0553	0.2912	0.0939	0.0751	X	
**Father's occupation**	0.0237	0.17	0.0972	0.0927	0.1891	X

**Table 3:** Determinants for complete Penta-3 vaccination of study participants, children aged 4 to 11 months, in urban settlements of Karachi, Pakistan.

**Explanatory variable**	**Odds ratio**	**95 % CI**	**p-value**	**Adjusted odds ratio**	**95 % CI**	**p-value**
**Gender of child**						
• (ref male)						
• Female	0.92	0.76–1.11	0.384	0.89	0.73–1.08	0.22

**Ethnicity**						
• Pashto	(ref)					
• Non-Pashto	2.05	1.66–2.55	<0.001	1.69	1.33––2.14	<0.001

**Place of delivery**						
• Community-based	(ref)					
• Facility-based	1.2	0.99–1.43	0.063	1.01	0.83–1.24	0.87

**Distance to EPI centre**						
• <2km	(ref)					
• >2km	0.59	0.49–0.71	<0.001	0.62	0.51–0.76	<0.001

**Mother’s education status**						
• No education/Madarsa/ Home-based	(ref)					
• Primary	2.2	1.56–3.11	<0.001	1.99	1.40–2.84	<0.001
• Secondary and above	3.82	3.09–4.72	<0.001	2.95	2.34–3.71	<0.001

**Father’s occupation**						
• Unemployed/Daily Wagers	(ref)					
• Self-employed	1.2	0.97–1.50	0.111	1.19	0.94–1.52	0.149
• Formally employed	2.16	1.72–2.72	<0.001	1.53	1.19–1.97	<0.001

According to our findings, gender of a child did not significantly alter the likelihood of Penta-3 immunization. The odds of completing Penta-3 immunization were nearly higher for children from non-Pashto speaking families (aOR 1.69; 95 % CI 1.33–2.14) relative to Pashto-speaking families. Mother’s education was significantly linked to Penta-3 uptake. The odds of Penta-3 immunization was two times for children of mothers with primary education (aOR 1.99, 95 % CI 1.40–2.84) and three times for children of mothers with secondary or above (aOR 2.95, 95 % CI 2.34–3.71) against the reference of no formal maternal education.. Likelihood of Penta-3 immunization was higher for the children of formally employed fathers (OR 1.53; 95 % CI 1.19–1.97), relative to daily wagers or unemployed fathers.

In the adjusted model, the odds of vaccination do not increase significantly for facility-based births, relative to community-based deliveries (aOR 1.01, 95 % CI 0.83–1.24). Residing at a distance of more than 2 km from the government EPI centre was negatively associated with Penta-3 uptake (aOR 0.62; 95 % CI 0.51–0.76).

## Discussion

The study attempted to identify the predictors of Penta-3 immunization, taken as a proxy for age-appropriate vaccination, in children residing in urban slums of Karachi labelled as ‘high-risk’ for polio. Within this study population, only 31 % of the children had completed their scheduled immunization up to Penta-3, lower than previously reported province-wide figures of 59 % in PDHS 2017–18 and 73 % reported by the Third-Party Verification of Immunization Coverage Survey (TPVICS) [18], [19]. To the best of our knowledge, this is the first study reporting determinants of coverage of Pentavalent-3 vaccine from high-risk union-councils of Karachi, whereas previous publications have been based on mainstream population datasets from Pakistan [20], [21], [22]. Rate of refusal in our survey was minimal, with only 1 % refusing to participate.

Our study did not find an association between the child’s gender and vaccine uptake. This is in contrast to previous studies from Pakistan and India that found girls to be less likely to be fully immunized [6], [23] potentially due to male children being more valued in patriarchal societies. We observed differences between immunization coverage associated with ethnicity. A child belonging to a non-Pashtun family was nearly twice as likely to receive Penta-3 vaccine. This finding is particularly important in the context of Karachi, which is a complex convergence of multiple ethnicities with large numbers of Pashtun migrants. Earlier studies from mainstream Pakistan have also reported lower vaccination completion rates among Pashtun families, relative to other ethnic groups despite similar level of poverty, outlining the need for greater effort to overcome cultural barriers [24], [25].

Our study reports significantly greater likelihood of Penta-3 vaccination in children whose mothers have completed primary education or above. The odds of immunization further increase for secondary or higher levels of mother’s education. Several previous studies have reported mother’s education as an important determinant of childhood vaccination in low-middle income countries [26], [27]. It can hence be hypothesized that promoting female education can significantly enhance the coverage of vaccination.

In our study, paternal occupation was associated with uptake of Penta-3 vaccine – children whose fathers did not have a stable source of income as in the case of unemployed or daily wage earner fathers, were more likely to miss Penta-3 vaccination. This is similar to studies from urban India and Bangladesh which showed that children with fathers in a business or service had higher odds of getting vaccinated, relative to unemployed fathers or those who worked as manual labourers [28], [29].

Contrary to other studies that have reported institutional delivery to be a positive determinant for full childhood vaccination [29,30] our study reports no significant difference in the likelihood of vaccine uptake between institutional versus community-based births. This indicates critical missed encounters for vaccination within urban health facilities, being utilized for new-born deliveries. In addition, nearly three quarters of primary health care in Karachi’s slums, as elsewhere in Pakistan’s cities is catered by private health providers due to a deficient government PHC infrastructure [31]. On the other hand, distance from the health facility (measured in kms) was found to be a significant predictor of Penta-3 vaccination and aligns with other studies from India, Bangladesh where children residing closer to vaccination centres have shown higher likelihood of getting vaccinated [32], [26].

In summary, likelihood of missed vaccinations was seen amongst children of Pashtun ethnicity, and those whose mothers lack any formal education, fathers are either unemployed or are daily wage earners, and reside at a distance of more than 2 km from the vaccination centre. Gender and place of maternal delivery did not have an effect on missed vaccination.

Multi-pronged strategies involving targeted demand generation for these population sub-sets (Pashtuns, uneducated mothers, daily wager parents), bringing vaccination centres closer to these populations and plugging of missed opportunities at birthing facilities especially within private sector, are required to meet the challenge of rapidly growing urban slums.

### Limitations

Prior experience of healthcare service use, child’s birth order and father’s education were not included in the analysis. Both vaccination card and mother’s recall were used as evidence for vaccine uptake, whereas there might be inaccuracies associated with mother’s recall [33].

## Conclusion

Pockets of critically low under-vaccinations within the urban slums of Karachi are associated with Pashtun ethnicity, lack of mothers’ education and father’s occupation as daily wagers. Recognition of these factors is required in designing contextually appropriate and multi-pronged strategies of targeted communication, brining vaccination centres closer to these populations and plugging missed opportunities during maternity care at health facilities.

## CRediT authorship contribution statement

**Shifa Salman Habib:** . **Shehla Zaidi:** Conceptualization, Funding acquisition, Investigation, Methodology, Project administration, Resources, Supervision, Validation, Visualization, Writing – review & editing. **Atif Riaz:** Investigation, Methodology, Project administration. **Hasan Nawaz Tahir:** . **Lala Aftab Mazhar:** Project administration, Supervision. **Zahid Memon:** Conceptualization, Investigation, Project administration.

## Declaration of competing interest

The authors declare that they have no known competing financial interests or personal relationships that could have appeared to influence the work reported in this paper.

## Data Availability

Data will be made available on request.
